# Anti-*Trichophyton* Activity of Protocatechuates and Their Synergism with Fluconazole

**DOI:** 10.1155/2014/957860

**Published:** 2014-06-18

**Authors:** Luciana Arantes Soares, Fernanda Patrícia Gullo, Janaina de Cássia Orlandi Sardi, Nayla de Souza Pitangui, Caroline Barcelos Costa-Orlandi, Fernanda Sangalli-Leite, Liliana Scorzoni, Luis Octávio Regasini, Maicon Segalla Petrônio, Patrícia Fernanda Souza, Dulce Helena Siqueira Silva, Maria José Soares Mendes-Giannini, Ana Marisa Fusco-Almeida

**Affiliations:** ^1^Laboratory of Clinical Mycology, Department of Clinical Analysis, Faculty of Pharmaceutical Sciences, UNESP, Rodovia Araraquara-Jaú, Km 1, 14801-902 Araraquara, SP, Brazil; ^2^Institute of Chemistry, UNESP, Rua Professor Francisco Degni 55, 14800-900 Araraquara, SP, Brazil; ^3^Department of Clinical Mycology, Faculty of Pharmaceutical Sciences, Universidade Estadual Paulista (UNESP), Rodovia Araraquara-Jaú, Km 1, 14801-902 Araraquara, SP, Brazil

## Abstract

Dermatophytosis and superficial mycosis are a major global public health problem that affects 20–25% of the world's population. The increase in fungal resistance to the commercially available antifungal agents, in conjunction with the limited spectrum of action of such drugs, emphasises the need to develop new antifungal agents. Natural products are attractive prototypes for antifungal agents due to their broad spectrum of biological activities. This study aimed to verify the antifungal activity of protocatechuic acid, 3,4-diacetoxybenzoic, and fourteen alkyl protocatechuates (3,4-dihydroxybenzoates) against *Trichophyton rubrum* and *Trichophyton mentagrophytes* and to further assess their activities when combined with fluconazole. Susceptibility and synergism assays were conducted as described in M38-A2 (CLSI), with modifications. Three strains of *Trichophyton rubrum* and three strains of *Trichophyton mentagrophytes* were used in this work. The pentyl, hexyl, heptyl, octyl, nonyl, and decyl protocatechuates showed great fungicidal effects, with minimum inhibitory concentration (MIC) values ranging from 0.97 to 7.8 mg/L. Heptyl showed a synergistic activity (FIC index = 0.49), reducing the MIC of fluconazole by fourfold. All substances tested were safe, especially the hexyl, heptyl, octyl, and nonyl compounds, all of which showed a high selectivity index, particularly in combination with fluconazole. These ester associations with fluconazole may represent a promising source of prototypes in the search for anti-*Trichophyton* therapeutic agents.

## 1. Introduction

Superficial fungal infections are a major global public health problem that affects 20–25% of the population worldwide [1]. Among these diseases, dermatophytosis, or tinea, is one of the most frequent fungal infections. This infection is caused by dermatophyte species that belong to the* Trichophyton, Microsporum, *or* Epidermophyton *genera [2]. These dermatophytes commonly invade different keratinophilic regions of the body, causing tinea corporis, tinea cruris, tinea pedis, tinea manus, tinea capitis, tinea barbae, and tinea unguium [3]. Dermatophyte infections can lead to either mild or severe symptoms, depending on the immunological response of the host [4]. Several patient groups also seem to be especially at risk of infection, including individuals with uncontrolled diabetes, AIDS, renal diseases, psoriasis, and types of immunosuppression, such as transplant recipients and patients on long-term corticosteroid therapy [5].

There is an urgent need to find new sources of substances with antidermatophytic activity because the treatment of dermatophytosis is long and expensive, particularly in the case of onychomycosis. Furthermore, the spectrum of the available drugs is limited, such drugs may induce adverse effects, and several reports of antifungal resistance have been published [6–9]. For this reason, various antifungal agents have been introduced into clinical practice, among them, amorolfine, ciclopirox, griseofulvin, terbinafine, itraconazole, fluconazole, and more recently, voriconazole [10, 11]. However, efforts should be concentrated on the discovery and development of novel, safer, and effective antidermatophytic agents.

Protocatechuic acid (3,4-dihydroxybenzoic) is a phenolic compound produced by the secondary metabolism of plants. It is naturally present in almost all plant materials, including food, fruits, and vegetables [12–14]. Together with its natural and synthetic derivatives, it has been associated with a broad spectrum of biological actions and is known to have antioxidant, proapoptotic [15], anti-inflammatory, antiglycative [16], and antimelanogenic [17] functions. However, the major interest in protocatechuic acid and its derivatives is due to its antimicrobial properties. It has been reported that protocatechuic acids have activity against susceptible and antibiotic-resistant* Campylobacter *spp. and* Helicobacter pylori* [18, 19]. Furthermore, it has been shown that *n*-octyl 3,4-dihydroxybenzoate has fungicidal activity against* Saccharomyces cerevisiae *[20].

Thus, considering the broad spectrum of protocatechuates and the need for the discovery of new antifungal agents, this study aimed to investigate the antifungal activity of a synthetic homologous series of* n*-alkyl protocatechuates against* Trichophyton rubrum* and* Trichophyton mentagrophytes*. This study also aimed to investigate the chemical characteristics of the compounds responsible for the biological activity of dermatophytes, including the importance of free hydroxyl radicals and the size of the carbon side chain.

## 2. Methods

### 2.1. Compounds Synthesis

Synthetic compounds of protocatechuic acid were prepared as described by de Faria et al. [21], with minor modifications. Briefly, a 3 mL solution of* N*,* N*-dicyclohexylcarbodiimide (DCC, 1 mmol) in *p*-dioxane was added to a cooled (5°C) solution of 0.2 mmol protocatechuic acid (**1**) (Sigma-Aldrich, St. Louis, MO, USA) and 20 mmol of *n*-alkyl alcohols in 6 mL of *p*-dioxane. The solution was stirred for 48 h and the solvent was removed under reduced pressure. The residue was partitioned 3 times with EtOAc and filtered. The filtrate was washed successively with a saturated aqueous citric acid solution (3 times) and saturated aqueous NaHCO_3_ (3 times), dried over anhydrous MgSO_4_, and evaporated under reduced pressure. The crude products were purified over a silica gel column (0.06–0.20 mm, ACROS Organics, USA) and eluted isocratically with CHCl_3_/MeOH (98 : 2) to produce esters** 2**−**15**. Their structures were then established by ^1^H and ^13^C NMR spectral analysis. For the synthesis of compound** 16**, protocatechuic acid (20 mmol) was dissolved in dried pyridine (5.0 mL) and anhydride acetic (5.0 mL) under a hydrogen atmosphere. The mixture was then stirred for 48 h at room temperature, dried under reduced pressure, and purified by column chromatography with a mixture of CHCl_3_/MeOH (85 : 15) to produce product** 16**. The NMR spectroscopic data for compound** 16** were compatible with it being 3,4-diacetoxybenzoic acid.

### 2.2. Microorganisms

To evaluate its antifungal activity, six species of dermatophytes were tested: two clinical strains of* Trichophyton rubrum* (Tr1 and Tr2),* Trichophyton rubrum* ATCC MYA 3108, two clinical strains of* Trichophyton mentagrophytes* (Tm1 and Tm2), and* Trichophyton mentagrophytes* ATCC 40131 (Tm3). The microorganisms were obtained from the collection of the Clinical Mycology Laboratory of the Department of Clinical Analyses at the School of Pharmaceutical Sciences of Universidade Estadual Paulista (UNESP). The strains were cultivated on Sabouraud dextrose agar (Difco, BD Biosciences) and incubated at 28°C for 7–15 days. For all experiments, the strains were cultivated on Potato Dextrose Agar (Difco, BD Biosciences) and incubated at 28°C as described above or until sporulation.

### 2.3. Dilution of Test Substances

The dilution of the synthetic compounds was performed with DMSO (Synth, Diadema, Sao Paulo, Brazil) as described by Scorzoni et al. [22]. The concentrations of the compounds on 96-well plates (TPP, Trasadigen, Switzerland) ranged from 500 mg/L to 0.97 mg/L. The antifungal drugs were diluted according to the CLSI M38-A2 document [23]. Stock solutions of Fluconazole (Sigma-Aldrich, St. Louis, MO, USA) were prepared, considering its power. Serial twofold dilutions were prepared according to the recommendations of Zhang and collaborators [24], with some modifications.

### 2.4. Minimum Inhibitory Concentration (MIC)

The antifungal activity tests were performed using the broth microdilution method as described in M38-A2, a document produced by the Clinical and Laboratory Standards Institute (CLSI, 2008) [23], with modifications. The medium used was RPMI 1640 with L-glutamine (Sigma-Aldrich, St. Louis, MO, USA) buffered to pH 7.0 with 0.165 M morpholinepropanesulfonic acid (MOPS) (Sigma-Aldrich, St. Louis, MO, USA), supplemented with 2% glucose. The cell suspension was prepared in a 0.85% saline solution. The suspension of conidia was then transferred to small sterile test tubes where they remained for 40 minutes to separate the microconidia, which were lighter and therefore present in the supernatant. The separated microconidia were then counted with a hematocytometer, and their concentration was adjusted to obtain a final concentration ranging from 2.5 × 10^3^ to 5 × 10^3^ CFU/mL. These suspensions were diluted in RPMI 1640 (Sigma-Aldrich, St. Louis, MO, USA) and inoculated on 96-well plates (TPP, Trasadigen, Switzerland) that had been previously prepared with the compounds diluted at concentrations from 250 to 0.48 mg/L. Positive (100 *μ*L of RPMI medium with 100 *μ*L of inoculum) and negative (200 *μ*L of RPMI) controls were included in all experiments. The plates were incubated with agitation at 35°C for 7 days. The MIC reading was performed by spectrophotometry at 490 nm. For fluconazole, the MIC was defined as the concentration that produced a 50% inhibition of fungal growth.

### 2.5. Minimum Fungicide Concentration (MFC)

A qualitative analysis of the fungal viability was performed by transferring a portion of the wells to a plate with Sabouraud (Difco, BD Biosciences) medium and incubating it at 35°C for the time determined for each fungal agent. The MFC was defined as the lowest extract concentration that did not allow the growth of any fungal colonies on the solid medium after the incubation period [25]. A visual reading was performed to confirm the death or growth inhibition provided by fluconazole and the sixteen semisynthetic substances derived from protocatechuic acid.

### 2.6. Synergistic Activity

The drug activity was assessed using a checkerboard method derived from a standardised procedure established by the National Committee for Clinical Laboratory Standards (M38-A2) [23]. Briefly, the test was performed on the same medium used for susceptibility testing. Volumes of 50 *μ*L of each drug in a concentration four times the final concentration were dispensed in 96-well plates (TPP, Trasadigen, Switzerland). To each well, 100 *μ*L of the fungal suspension was added to produce a final concentration of 5.0 × 10^3^ CFU/mL.

As a negative control, we used 200 *μ*L of RPMI, while as a positive control, we used 100 *μ*L of RPMI medium with 100 *μ*L of inoculum. The plates were incubated at 35°C, and the reading was completed after 168 hours. We conducted visual and spectrophotometric readings at 490 nm. To determine the effect of combinatorial fractions, we calculated the fractional inhibitory concentration (FIC). The FIC was calculated by taking the MIC of the substance in combination/MIC of the substance alone. The sum of the fractional inhibitory concentration (FIC) of each substance consists of the fractional inhibitory concentration index: (the MIC of drug A in combination/the MIC of drug A alone) + (the MIC of drug B in combination/the MIC of drug B alone). A synergistic relationship was defined as FIC index ≤0.5, an additive relationship was defined as 0.5 < FIC index ≤ 1.0, an indifferent relationship was defined as 1.0 < FIC index ≤ 4.0, and an antagonistic relationship was defined as FIC index >4.0 [26–28].

### 2.7. Cytotoxicity Assay

The cytotoxicity of sixteen semisynthetic compounds derived from protocatechuic acid was assessed using a sulphorhodamine B assay in NOK (oral human keratinocyte) cell lines obtained from the American Type Culture Collection (Manassas, VA, USA). The strains were maintained in bottles appropriate for cell culture with keratinocyte serum free medium (Gibco, Life Technologies) and incubated in standard conditions of 37°C and 5% CO_2_. Cell concentrations ranging from 2.5 to 5.0 × 10^4^ cells/mL were used for the formation of cell monolayers. The concentrations of pure substances were kept in contact with the cells for 24 hours. After the incubation period, the cells were treated with the sulphorhodamine B reagent (Sigma-Aldrich, St. Louis, MO, USA) as previously described by Skehan et al. [29], with some modifications.

### 2.8. Statistical Analysis

All experiments were performed in triplicate. Statistical analysis was performed with a* t*-test or one-way ANOVA with GraphPad Prism 5 software (Version 5, USA). *P* values < 0.05 were considered statistically significant.

## 3. Results

### 3.1. Antidermatophytic Activity

Protocatechuic acid, 3.4 diacetoxibenzoic acid, and fourteen-alkyl protocatechuate (3,4-dihydroxybenzoate) derivatives were evaluated against 6 strains of* Trichophyton rubrum* and* Trichophyton mentagrophytes*. The minimum inhibitory concentration (MIC) ranged from ≥250 to 1.95 mg/L. Pentyl, hexyl, heptyl, octyl, nonyl, and decyl protocatechuate compounds showed the best MIC values, ranging from 1.95 to 7.8 mg/L for both species, with MFC values ranging from 1.95 to 15.6 mg/L and 0.97 to 15.6 mg/L, for* Trichophyton rubrum* and* Trichophyton mentagrophytes, *respectively (Table 1). In contrast, protocatechuic acid and acid 3.4 diacetoxybenzoate showed the highest values of MIC and MFC, from 125 to >250 mg/L, for both species and were thus considered to have low antidermatophytic activity. The potentiation of antifungal activity was observed with an increase of methylation in the structure of protocatechuic acid. However, the addition of nine methyl groups [(CH_2_) 9CH_3_] led to a progressive reduction in the MIC values. The addition of methyl groups produced a reduction in the MIC and MFC values for strains of both species. The addition of a methyl group (CH_3_) produced a range of MIC and MFC values of 31.25 to 62.50 mg/L for the isolates of* Trichophyton rubrum* and* Trichophyton mentagrophytes*. The only exception was for Tm1 because despite its MIC of 62.50 mg/L, its MFC remained at 250 mg/L. Ethyl and propyl showed MIC and MFC values ranging from 15.62 to 62.50 mg/L for both species of dermatophytes. The addition of four methyl groupings (pentyl protocatechuate) produced a strong antifungal activity, as the MIC values were low, ranging from 3.90 to 7.80 mg/L, while the MFC ranged from 3.90 to 15.60 mg/L. Therefore, dodecyl, tetradecyl, hexadecyl, and octadecyl showed low activities, with their MIC and MFC values ranging from 125 to >250 mg/L for* Trichophyton rubrum* strains and from 31.25 and >250 mg/L for* Trichophyton mentagrophytes* strains.

### 3.2. Synergistic Activity

The activity of the combination of fluconazole with pentyl, hexyl, heptyl, octyl, and nonyl protocatechuates was evaluated and classified as antagonistic, indifferent, additive, and synergistic based on the FIC index, as shown in Table 2. The FIC index was calculated based on the results of the checkerboard test.

The combinations were tested in clinical isolates of* Trichophyton rubrum* (Tr1) and* Trichophyton mentagrophytes* (Tm1) and in the reference strain* Trichophyton mentagrophytes* ATCC 40131 (Tm3).

For the clinical isolates of* Trichophyton rubrum* (Tr1), the MIC value of fluconazole was 2.0 mg/L. When associated with the protocatechuate pentyl (compound** 7**), a reduction in the MIC of fluconazole of 16.66 times (0.12 mg/L) (*P* < 0.05) and a conservation of the MIC of compound** 7** (7.8 mg/L) were observed. The same occurred when fluconazole was associated with protocatechuate heptyl (substance** 9**) (*P* < 0.05). Thus, both associations were classified as additive, with an FIC index of 1.0. The other acids tested showed a conservation of the fluconazole MIC (2.0 mg/L) (*P* > 0.05) and a reduction in the MIC value of protocatechuates. Compound** 8** (hexyl protocatechuate) reduced the MIC value by a factor of 8.125 (0.48 mg/L) (*P* < 0.05). Compound** 10** (octyl protocatechuate) reduced the MIC by a factor of 31.66 (0.06 mg/L) (*P* < 0.01), and compound** 11** (nonyl protocatechuate) reduced the MIC by 63.33 times (0.03 mg/L) (*P* < 0.01). Thus, these combinations were classified as additive, with an FIC index of 1.0. The only exception was compound** 8**, which had an FIC index of 1.1 and was thus classified as indifferent.

In the clinical isolate Tm1, the combination of fluconazole and all protocatechuates acids tested showed a conservation of the fluconazole MIC (1.0 mg/L) (*P* > 0.05) and a 130-fold reduction in the MIC values of the protocatechuates for substances** 7**,** 9, **and** 11** (0.03 mg/L) (*P* < 0.01) and in 63.33 times for compound** 8** and** 10 **(0.03 mg/L) (*P* < 0.01). All combinations were classified as additive for Tm1, with an FIC index value of 1.0. However, for the strain ATCC 40131 (Tm3), a conservation in the fluconazole MIC (0.5 mg/L) was observed when combined with compounds** 7** and** 11** (*P* > 0.05). The combination of the activity between these compounds was classified as additive, with an FIC index equal to 1.0. A reduction in the MIC values of two-, four-, and twofold, respectively, for substances** 8**,** 9,** and** 10 **(*P* < 0.05) was observed when combined with fluconazole. A fourfold reduction (0.12 mg/L) in the MIC value was observed when associated with these compounds (*P* < 0.05). The associations between fluconazole and compounds 8 and 10 were classified as additive and had an FIC index equal to 0.72, whereas the association with compound** 9** instead had a synergistic activity, with an FIC index equal to 0.48.

### 3.3. Cytotoxicity Assay

The compounds pentyl (**7**), hexyl (**8**), heptyl (**9**), octyl (**10**), and nonyl (**11**) were evaluated in a cytotoxicity assay in NOK cells. These substances showed high values for IC_50_ after reaching the necessary concentration to produce 50% lethality of 57 mg/L for cells treated with compound** 7**, 66.5 mg/L for cells treated with compound** 8**, 78.1 mg/L for cells treated with compound** 9**, 54 mg/L for cells treated with compound** 10,** and 52.1 mg/L for cells treated with compound** 11**. These results showed that these compounds had a low toxicity in human oral keratinocytes (Table 3). The selectivity index (SI) was calculated for all six strains of* Trichophyton rubrum* and* Trichophyton mentagrophytes*. A selectivity index greater than 10 indicates a substance with a higher selectivity for fungi. Considering all six strains, compounds** 9** and** 11** had SI values greater than 10. The SI values for compounds** 9** and** 11** ranged from 20.0 to 80.5 and 13.2 to 26.7, respectively.

## 4. Discussion

It is estimated that superficial mycoses affect approximately 25% of the world's population [30]. Due to the high rate of recurrence of superficial fungal infections and the increasing problem of antifungal resistance, especially against the azole family of drugs, new treatment alternatives with fungicidal activity are sorely needed [31–33]. In this context, the anti-*Trichophyton* spp. activity of protocatechuic acid, 3,4-diacetoxibenzoic acid, and fourteen alkyl protocatechuates (3,4-dihydroxybenzoates) were evaluated on their own or in combination with fluconazole. In the sixteen compounds studied, significant antifungal activity was found. Remarkable results were observed in six compounds against the two species with pentyl, hexyl, heptyl, octyl, nonyl, and decyl compounds. These results showed that increasing the length of the side chain by up to 9 carbons enhanced both the hydrophobicity and thus the antifungal activity of the compounds. However, the addition of more than 9 carbons leads to a reduction in the antifungal activity. The same protocatechuic acid esters were used by de Faria et al. [21]. However, in their work, the antioxidant activity of those compounds was evaluated. They also reported that the alkylation process increased the hydrophobicity of these compounds, resulting in an increased inhibition of the oxidative process. Nihei and collaborators [20] evaluated the series of alkyl 3,4-dihydroxybenzoates (protocatechuates) and their fungicidal activity against the yeast* Saccharomyces cerevisiae*. Nonyl and octyl, 3,4-dihydroxybenzoates obtained the lowest values of MIC. In addition, the anti-*Saccharomyces *activity was also correlated with the hydrophobicity of the carbon chain. Thus, by analysing the structure-activity relationship of these compounds, we can conclude that the carbon chain has a key role not only in antioxidant activity, as described by de Faria and collaborators [21], but also in the antifungal activity against* Trichophyton* spp.

Due to the lack of new classes of drugs or different molecular targets, drug combinations can be considered a strategy for therapy [34]. Several studies have proposed the use of natural compounds in combination with drugs to establish a new strategy for the treatment and prevention of certain diseases [35–38]. Different combinations of statins and some antifungal drugs were tested against four dermatophyte species (*Trichophyton mentagrophytes*,* Trichophyton rubrum*,* Microsporum canis*, and* Microsporum gypseum*). Most of the synergistic activity was found with the combination of statins with terbinafine and the different azoles [39]. Our results showed additive and synergistic activity of the protocatechuic acid derivatives with fluconazole against* Trichophyton rubrum* and* Trichophyton mentagrophytes*. We observed a synergistic activity of fluconazole associated with the heptyl derivative (**9**) when tested against Tm3, with a reduction in the MIC value of fluconazole and heptyl by four- and eightfold, respectively; an additive activity was observed for Tm1 and Tm3 with this combination. For the isolate Tr1, we observed additive activity with the association of pentyl, heptyl, octyl, and nonyl with fluconazole. For this isolate, the combination of a hexyl derivative (**8**) with this azole was found to not produce a change in its antifungal activity, such that this association was considered indifferent. All other combinations produced additive activity against the isolates tested. The great advantage of using phenolic compounds in combination with conventional therapies is that they can increase the susceptibly of microorganisms compared to the usual drugs and therefore are associated with reduced toxicity [38].

Palafox-Carlos and collaborators [40] reported an antioxidative synergistic effect between phenolic acids present in mango (*Mangifera indica* L.) and protocatechuic acid, gallic acid, vanillic acid, and chlorogenic acid. The authors found that the association showed a synergistic effect and that the gallic and protocatechuic acids presented higher antioxidative capacities. Jayaraman and collaborators [38] studied the combination of seven antibiotics and six phytochemical compounds, including protocatechuic acid, against isolates of* Pseudomonas aeruginosa* and found that the combination of protocatechuic acid and sulfamethoxazol showed synergistic activity against all bacterial isolates, with a fractional inhibitory concentration index of 0.25 to 0.5.

The efficacy of fluconazole can be improved using combination therapy [34]. Aala and collaborators [41] found good activity between allicin in combination with ketoconazole or fluconazole demonstrating a synergistic or additive interaction against dermatophytes. Galgóczy and coauthors [42] evaluated the* in vitro* antifungal activity against strains of dermatophytes, combining the protein (PAF) of* Penicillium chrysogenum* with fluconazole (FCZ). PAF and FCZ acted synergistically and/or additively on all of the tested fungi except* Microsporum gypseum*, for which no interactions were detected [42].

When the cytotoxicity of the substances was evaluated, high IC_50_ values were found. The IC_50_ indicates the concentration of the compound that is necessary to kill 50% of the cell line. The selective index was also calculated using the ratio of the IC_50_ and MFC. For all of the isolates of the two species tested, most IS values of the substances were higher than 10, and the best results were found for the heptyl and nonyl derivatives. The IS values indicate that the concentrations tested caused injury to the fungal cells but no toxicity to the human cells [43–45].

Compounds not recognised as antifungal agents may cause potent inhibition of growth when used in combination with fluconazole. Our results suggest that the combination of substances can act with synergistic or additive activity in the treatment of dermatomycosis. More studies related to these combinations should be performed to identify a possible mechanism of action and to provide a verification of their* in vivo* activity. The evaluation of drug combinations defines a strategy for the discovery of new therapies, leading to a future clinical evaluation. In addition to the potent antidermatophytic capabilities, the protocatechuate derivatives presented a low toxicity to keratinocytes, demonstrating that the topical use of these novel compounds may represent a promising new option for the treatment of superficial mycoses.

## 5. Conclusion

In summary, new therapeutic trials are important to unravel biological data and thus result in the discovery of new combination drug. Overall, our results may contribute to a database of information on test susceptibility and synergism of dermatophytes* in vitro*, targeting the development and optimization of antifungal drugs. In the present work the esters of protocatechuic acid as well as their associations with commercial drug could be a promising therapeutic approach. More studies are needed to determine the mechanism of action of these substances providing the molecular basis of understanding.

## Figures and Tables

**Table 1:** MIC and MFC values (mg/L) and quantitative analysis of fungal cellular viability of protocatechuic acid derivates against *Trichophyton *spp.

	R	Tr1	Tr2	Tr3	Tm1	Tm2	Tm3
		MIC (MFC)	MIC (MFC)	MIC (MFC)	MIC (MFC)	MIC (MFC)	MIC (MFC)
Acid protocatechuic	H	250 (250)	250 (250)	250 (250)	250 (>250)	250 (>250)	125 (125)
Acid 3.4 diacetoxybenzoic	—	250 (250)	>250 (>250)	>250 (>250)	250 (250)	>250 (>250)	250 (250)
Protocatechuate methyl	CH_3_	62.5 (62.5)	31.2 (31.2)	31.2 (62.5)	62.5 (250)	62.5 (62.5)	31.2 (62.5)
Protocatechuate ethyl	CH_2_CH_3_	31.2 (31.2)	31.2 (31.2)	31.2 (31.2)	31.2 (62.5)	31.2 (31.2)	31.2 (125)
Protocatechuate propyl	(CH_2_)2CH_3_	15.6 (15.6)	31.2 (31.2)	31.2 (31.2)	15.6 (62.5)	62.5 (62.5)	15.6 (31.2)
Protocatechuate butyl	(CH_2_)3CH_3_	15.6 (15.6)	7.80 (7.80)	7.80 (7.80)	7.80 (62.5)	15.6 (15.6)	15.6 (15.6)
Protocatechuate pentyl	(CH_2_)4CH_3_	**7.80 (7.80)**	**7.80 (7.80)**	**7.80 (7.80)**	**3.90 (15.6)**	**7.80 (7.80)**	**7.80 (7.80)**
Protocatechuate hexyl	(CH_2_)5CH_3_	**3.90 (3.90)**	**1.95 (3.90)**	**3.90 (3.90)**	**1.95 (3.90)**	**3.90 (3.90)**	**3.90 (7.80)**
Protocatechuate heptyl	(CH_2_)6CH_3_	**1.95 (1.95)**	**1.95 (1.95)**	**1.95 (1.95)**	**3.90 (3.90)**	**0.97 (0.97)**	**3.90 (3.90)**
Protocatechuate octyl	(CH_2_)7CH_3_	**1.95 (1.95)**	**7.80 (7.80)**	**0.97 (1.95)**	**1.95 (3.90)**	**1.95 (1.95)**	**3.90 (3.90)**
Protocatechuate nonyl	(CH_2_)8CH_3_	**1.95 (1.95)**	**3.90 (3.90)**	**1.95 (3.90)**	**3.90 (3.90)**	**3.90 (3.90)**	**3.90 (3.90)**
Protocatechuate decyl	(CH_2_)9CH_3_	**3.90 (3.90)**	**3.90 (3.90)**	**3.90 (7.8)**	**7.80 (7.80)**	**3.90 (3.90)**	**3.90 (15.6)**
Protocatechuate dodecyl	(CH_2_)11CH_3_	>250 (>250)	125 (125)	>250 (250)	250 (250)	62.5 (62.5)	31.2 (62.5)
Protocatechuate tetradecyl	(CH_2_)13CH_3_	>250 (>250)	125 (125)	>250 (>250)	>250 (>250)	125 (250)	>250 (>250)
Protocatechuate hexadecyl	(CH_2_)15CH_3_	>250 (>250)	>250 (>250)	>250 (>250)	>250 (>250)	>250 (>250)	>250 (>250)
Protocatechuate octadecyl	(CH_2_)17CH_3_	>250 (>250)	>250 (>250)	>250 (>250)	>250 (>250)	>250 (>250)	>250 (>250)

MIC: minimal inhibitory concentration; MFC: minimal fungicidal concentration; Tr1 and Tr2: *T. rubrum* clinical isolates; Tr3: *T. rubrum* ATCC MYA 3108; Tm1 and Tm2: *T.  interdigitale* clinical isolates; and Tm3:* T. mentagrophytes *ATCC 40131.

**Table 2:** Activity of fluconazole combined with pentyl, hexyl, heptyl, octyl, and nonyl protocatechuates against *T. rubrum* and *T. mentagrophytes* (mg/L).

	MIC (mg/L)											FIC index (association type)				
	Substances alone						Substances combined									
	FLU	**7**	**8**	**9**	**10**	**11**	FLU + **7**	FLU + **8**	FLU + **9**	FLU + **10**	FLU + **11**	FLU + **7**	FLU + **8**	FLU + **9**	FLU + **10**	FLU + **11**
Tr1	2.0	7.8	3.9	1.9	1.9	1.9	0.12^∗^—7.80	2.00—0.48^∗^	0.12^∗^—1.9	2.00—0.06^∗^^∗^	2.00—0.03^∗^^∗^	1.0 (A)	1.1 (I)	1.0 (A)	1.0 (A)	1.0 (A)
Tm1	1.0	3.9	1.9	3.9	1.9	3.9	1.00—0.03^∗^^∗^	1.00—0.03^∗^^∗^	1.00—0.03^∗^^∗^	1.00—0.03^∗^^∗^	1.00—0.03^∗^^∗^	1.0 (A)	1.0 (A)	1.0 (A)	1.0 (A)	1.0 (A)
Tm3	0.5	7.8	3.9	3.9	3.9	3.9	0.50—0.03	0.12—1.9^∗^	0.12—0.97^∗^	0.12—1.9^∗^	0.50—0.03	1.0 (A)	0.72 (A)	0.48 (S)	0.72 (A)	1.0 (A)

MIC: minimal inhibitory concentration; FIC: fractional inhibitory concentration; S: synergistic effect; A: additive effect; I: indifferent effect Tr1: clinical strain of *T. rubrum*; Tm1: clinical strain of *T. mentagrophytes*; and Tm3: *T. mentagrophytes *ATCC 40131. ^∗^ *P* < 0.05; ^∗^^∗^ *P* < 0.01.

**Table 3:** Evaluation of IC_50_ and selectivity index of pentyl, hexyl, heptyl, octyl, and nonyl in NOK cells.

	Substances	Cytotoxicity IC_50_ (mg/L)	SI (IC_50_/MFC)					
			Tr1	Tr2	Tr3	Tm1	Tm2	Tm3
**7**	Pentyl	57.0	7.3	7.3	7.3	3.7	7.3	7.3
**8**	Hexyl	66.5	**17.0**	**17.0**	**17.0**	**17.0**	**17.0**	8.5
**9**	Heptyl	78.1	**40.0**	**40.0**	**40.0**	**20.0**	**80.5**	**20.0**
**10**	Octyl	54.0	**27.7**	7.0	**27.7**	**13.8**	**27.7**	**13.8**
**11**	Nonyl	52.1	**26.7**	**13.2**	**13.2**	**13.2**	**13.2**	**13.2**
